# Angiotensin-converting enzyme 2 is reduced in Alzheimer’s disease in association with increasing amyloid-β and tau pathology

**DOI:** 10.1186/s13195-016-0217-7

**Published:** 2016-11-25

**Authors:** Patrick Gavin Kehoe, Steffenny Wong, Noura AL Mulhim, Laura Elyse Palmer, J. Scott Miners

**Affiliations:** Dementia Research Group, University of Bristol, Level 1, Learning and Research, Southmead Hospital, Bristol, BS10 5NB UK

**Keywords:** Angiotensin-converting enzyme-2, Renin-angiotensin system, Angiotensin-converting enzyme-1, Angiotensin II, Alzheimer’s disease

## Abstract

**Background:**

Hyperactivity of the classical axis of the renin-angiotensin system (RAS), mediated by angiotensin II (Ang II) activation of the angiotensin II type 1 receptor (AT1R), is implicated in the pathogenesis of Alzheimer’s disease (AD). Angiotensin-converting enzyme-2 (ACE-2) degrades Ang II to angiotensin 1–7 (Ang ﻿﻿(1-7)) and counter-regulates the classical axis of RAS. We have investigated the expression and distribution of ACE-2 in post-mortem human brain tissue in relation to AD pathology and classical RAS axis activity.

**Methods:**

We measured ACE-2 activity by fluorogenic peptide substrate assay in mid-frontal cortex (Brodmann area 9) in a cohort of AD (*n* = 90) and age-matched non-demented controls (*n* = 59) for which we have previous data on ACE-1 activity, amyloid β (Aβ) level and tau pathology, as well as known *ACE1* (rs1799752) indel polymorphism, apolipoprotein E (*APOE*) genotype, and cerebral amyloid angiopathy severity scores.

**Results:**

ACE-2 activity was significantly reduced in AD compared with age-matched controls (*P* < 0.0001) and correlated inversely with levels of Aβ (*r* = −0.267, *P* < 0.001) and phosphorylated tau (p-tau) pathology (*r* = −0.327, *P* < 0.01). ACE-2 was reduced in individuals possessing an *APOE* ε4 allele (*P* < 0.05) and was associated with *ACE1* indel polymorphism (*P* < 0.05), with lower ACE-2 activity in individuals homozygous for the *ACE1* insertion AD risk allele. ACE-2 activity correlated inversely with ACE-1 activity (*r* = −0.453, *P* < 0.0001), and the ratio of ACE-1 to ACE-2 was significantly elevated in AD (*P* < 0.0001). Finally, we show that the ratio of Ang II to Ang (1–7) (a proxy measure of ACE-2 activity indicating  conversion of Ang II to Ang (1–7)) is reduced in AD.

**Conclusions:**

Together, our findings indicate that ACE-2 activity is reduced in AD and is an important regulator of the central classical ACE-1/Ang II/AT1R axis of RAS, and also that dysregulation of this pathway likely plays a significant role in the pathogenesis of AD.

**Electronic supplementary material:**

The online version of this article (doi:10.1186/s13195-016-0217-7) contains supplementary material, which is available to authorized users.

## Background

Genetic, clinical and epidemiological data as well as experimental cell and animal studies all support a role for the renin-angiotensin system (RAS) in the pathogenesis of Alzheimer’s disease (AD) [1]. Many of the pro-inflammatory, anti-cholinergic and vasopressor actions of RAS associated with the pathogenesis of AD are mediated by angiotensin II (Ang II) signalling through the angiotensin II type 1 receptor (AT1R), commonly referred to as the *classical axis* (reviewed in [1]). Intracerebroventricular infusion of Ang II increased both amyloid-β (Aβ) (via increased amyloidogenic processing of amyloid precursor protein [APP]) [2] and tau pathology, and also reduced cognitive performance [3], in aged normal rats. We have previously reported that angiotensin-converting enzyme-1 (ACE-1), the rate-limiting enzyme in the production of angiotensin II (Ang II), is increased in AD in human brain tissue [4, 5]. Angiotensin II type 1 receptor blockers (ARBs) and angiotensin-converting enzyme inhibitors (ACEIs) reduce the amount of AD-like pathology and improve cognitive performance in most but not all mouse models of AD [6–11]. Translation of these treatments in AD is also supported in secondary outcomes of clinical trials of various ARBs and ACEIs, as well as in epidemiological studies where the prevalence of AD was reduced [12–16]. Last, the *ACE-1* indel polymorphism (rs1799752) is a genetic risk factor for sporadic AD [17]. This finding has previously been supported by several meta-analyses [18–22] but not by recent genome-wide association studies.

ACE-2 is a zinc metallopeptidase which shares 42% sequence homology within the ACE-1 catalytic region [23, 24]. The ACE-2 metalloprotease is expressed mostly as a transmembrane protein, but it also exists in an active soluble truncated form [24]. It is expressed predominantly in endothelial and arterial smooth muscle cells throughout the body [25], but it is also expressed in non-vascular cells within the brain, including neuronal cell bodies [26] and astroglial cells [27]. Upon its discovery, ACE-2 was shown to generate angiotensin 1–7 (Ang (1-7)) from Ang II, and, to a lesser extent, angiotensin 1–9 (Ang (1-9)) from Ang I [23, 24, 28]. Emerging data suggest that ACE-2-mediated conversion of Ang II to Ang (1–7) and subsequent activation of the Mas receptor by Ang (1–7) (comprising the ACE-2/Ang (1-7) /Mas axis) oppose the local actions of the classical RAS pathway in both the periphery (reviewed in [29]) and brain (reviewed in [30–33]). In experimental animal studies, ACE-2 regulates blood pressure by counteracting the effects of the classical axis. A reduction in ACE-2 expression has been implicated in cardiac and renal pathologies (reviewed in [30]) associated with chronic hypertension. Activation of brain ACE-2 has been shown to be neuroprotective in animal models of ischaemic stroke [34, 35].

Previous studies have suggested a link between reduced activity of the ACE-2/Ang (1–7)/Mas axis and neurodegenerative conditions, including multiple sclerosis [36]. A recent study provided the first clues of an association with AD and reported reduced serum ACE-2 activity in patients with AD compared with control subjects [37]. Notably, this study also identified that ACE-2 converts Aβ_43_ (an early deposited and highly amyloidogenic form of Aβ that seeds plaque formation [38]) to Aβ_42_, which in turn is cleaved by ACE-1 to less toxic Aβ_40_ and Aβ_41_ species [37]. Ang (1–7) levels were also reduced in a mouse model of sporadic AD in association with hyperphosphorylation of tau [39].

In the present study, we investigated the expression and distribution of ACE-2 in relation to AD pathology and the classical RAS axis in human post-mortem brain tissue. We show, for the first time to our knowledge, that ACE-2 activity is reduced in human post-mortem brain tissue in AD in relation to Aβ and tau pathology, and also that ACE-2 correlates inversely with ACE-1 activity. We also show that the ratio of Ang II to Ang (1–7) (a proxy measure of ACE-2 activity) was increased in AD, indicating reduced conversion of Ang II to Ang (1–7). Together, these data indicate that the ACE-2/Ang (1–7)/Mas axis is dysregulated in AD and that loss of function of this regulatory arm of RAS may contribute, at least in part, to overactivation of the classical RAS axis associated with AD pathogenesis.

## Methods

### Case selection

Brain tissue was obtained from the South West Dementia Brain Bank, University of Bristol, UK, with local research ethics committee approval (National Research Ethics Service 08/H0106/28 + 5). Tissue was dissected from the mid-frontal cortex (Brodmann area 9) in 90 cases of AD and 59 age-matched controls. Brains had been subjected to detailed neuropathological assessment according to the National Institute on Aging-Alzheimer’s Association guidelines [40], and AD pathology was a sufficient explanation for the dementia in these cases. Control brains were from people who had no history of dementia, had been extensively assessed neuropathologically, and had few or absent neuritic plaques, Braak tangle stage III or less, and no other neuropathological abnormalities. The demographic data for these cases are presented in Table 1, and the Medical Research Council UK Brain Banks Network (MRC UK-BBN) database identifiers are shown in Additional file 1: Table S1.

**Table 1:** Demographics of the study cohort

	Control (*n* = 59)	AD (*n* = 90)
Age, years, mean ± SD	78.5 ± 10.1	78.5 ± 9.7
Sex, F/M	22/37	55/35
PM delay, h, mean ± SD)	43.8 ± 36.4	45.2 ± 25.1

*AD* Alzheimer’s disease, *PM* Post-mortem

Previous measurements of ACE-1 activity, measured by fluorogenic activity assay, were available for all cases [4, 41]. Total soluble (Nonidet P-40-extracted) and insoluble (6 M guanidine hydrochloride-extracted) Aβ levels were measured previously by sandwich enzyme-linked immunosorbent assay (ELISA) [42], and cerebral amyloid angiopathy (CAA) severity, which was graded semi-quantitatively on a 4-point scale by a method adapted from that of Olichney et al. [43], had previously been reported [44]. Phosphorylated tau (p-tau) load (area fraction of cerebral cortex immunopositive for p-tau) had been measured for all cases, as previously reported [45, 46]. *ACE1* genotype data for the Alu 237-bp insertion(I)/deletion(D) (indel) polymorphism (rs1799752) in intron 16 of the ACE1 gene were previously reported [5, 41]. Last, all cases had previously been apolipoprotein E (*APOE*)-genotyped [44, 47] by a polymerase chain reaction method [48].

### Brain tissue

The right cerebral cortex had been fixed in 10% formalin for a minimum of 3 weeks before the tissue was processed and paraffin blocks were taken for pathological assessment. The left cerebral hemisphere had been sliced and frozen at −80 °C until used for biochemical assessment. For each case, 200 mg of dissected frozen brain tissue was homogenised in a Precellys homogeniser (Stretton Scientific, Stretton, UK) as previously described [4, 5]. The samples were centrifuged at 13,000 rpm, and the clarified supernatants were aliquoted and stored at −80 °C until required. Total protein was measured using the Total Protein kit (Sigma-Aldrich, Poole, UK) following the manufacturer’s guidelines. All brain tissue was obtained within 72 h after death.

### ACE-2 activity assay

ACE-2 activity was measured in brain tissue using the SensoLyte® 390 ACE2 activity assay kit (catalogue number AS-72086; AnaSpec, Fremont, CA, USA). The assay was performed in black, flat-bottomed, non-binding, 96-well Nunc FluoroNunc plates (Fisher Scientific, Loughborough, UK) following the manufacturer’s guidelines with minor modifications. Brain tissue homogenates were prepared in assay buffer provided in the kit, to which 0.05% Triton X-100 was added. Samples were centrifuged at 13,000 rpm for 15 minutes at 4 °C, and supernatants were removed and stored at −80 °C until used. Supernatants were diluted 1:100 in the proprietary ACE-2 assay buffer and incubated for 10 minutes at room temperature prior to addition of the ACE-2-specific fluorescence resonance energy transfer (FRET) peptide and then incubated for 30 minutes in the dark. Cleavage of the ACE-2 FRET peptide was measured using a BMG FLUOstar OPTIMA microplate reader (BMG Labtech, Aylesbury, UK) at an excitation/emission wavelength of 330/390 nm. ACE-2 activity was interpolated from a serial dilution of 7-methoxycoumarin-4-yl-acetyl (Mca) fluorescence reference standard, and measurements for each case were repeated in duplicate.

To confirm the specificity of the commercial ACE-2 assay kit, we measured ACE-2 activity in a subset of samples (ten controls and ten AD) for which we had previously measured ACE-2 activity as outlined above. The assay was performed in black, flat-bottomed, non-binding, 96-well Nunc FluoroNunc plates. Recombinant human ACE-2 (440-6 ng/ml) (R&D Systems, Cambridge, UK) and brain tissue supernatants (diluted 1:20) were diluted in assay buffer (75 mM Tris, 1 M NaCl, pH 7.5) and pre-incubated with an ACE-2 specific inhibitor, MLN4760 (10 μM) (Calbiochem, Nottingham, UK) or assay buffer alone for 10 minutes at 37 °C. An ACE-2 fluorogenic peptide Mca-APK(Dnp) (Enzo Life Sciences, Exeter, UK) was then added, and the reaction was incubated at 37 °C for 30 minutes in the dark. Fluorescence was read at an excitation/emission wavelength of 330/405 nm using a BMG FLUOstar OPTIMA microplate reader. ACE-2-specific activity was calculated after subtracting fluorescence in the presence of MLN-4760 from the uninhibited sample. We observed a very strong correlation between the independent measurements of ACE-2 in the presence of MLN4760 (10 μM) and with the kit, indicating the specificity of the ACE-2 assay kit (Additional file 2: Figure S1).

### Angiotensin II sandwich ELISA

Ang II levels were measured in brain tissue homogenates extracted in 1% SDS lysis buffer (100 μM NaCl, 10 mM Tris, pH 6, 1 μM phenylmethylsulphonylfluoride, 1 μg/ml aprotinin [Sigma-Aldrich] and 1% SDS in distilled water) using a commercially available sandwich ELISA kit (Abcam, Cambridge, UK) following the manufacturer’s guidelines. In brief, recombinant human Ang II or brain tissue supernatants (diluted 1:2 in PBS) were added in duplicate to wells that had been pre-coated with an Ang II-specific capture antibody and incubated for 2 h at room temperature. After a wash step, the wells were incubated for 2 h with biotinylated anti-Ang II antibody at room temperature. The plate was again washed, followed by a 30-minute incubation with streptavidin/HRP. After a final wash, 3,3′,5,5′-tetramethylbenzidine (TMB) substrate was added for 20 minutes, and the absorbance at 450 nm was read using a FLUOstar OPTIMA plate reader. The concentration of Ang II was interpolated from a serial dilution of recombinant Ang II (1000–62.5 pg/ml) and measured in duplicate for each case.

### Angiotensin (1–7) direct ELISA

Ang (1–7) levels were measured in human brain tissue homogenates in 1% SDS lysis buffer (see above) using an in-house direct ELISA kit. Recombinant human Ang (1–7) (Enzo Life Sciences) or human brain tissue homogenates (diluted 1:40 In PBS) were incubated for 2 h in a clear, high binding capacity Nunc MaxiSorp plate (Thermo Fisher Scientific, Waltham, MA, USA) at 26 °C with shaking. The wells were washed five times in PBS with 0.05% Tween-20 and blocked for 1 h in PBS:1% bovine serum albumin (Sigma-Aldrich). After another five washes, the wells were incubated with biotinylated anti-human Ang 1–7 (2 μg/ml in PBS) (Cloud-Clone, Wuhan, China) for 2 h at 26 °C with shaking, followed by a further wash step. Streptavidin/HRP (1:200) in PBS/0.01% Tween-20 was added to each well, which was incubated at room temperature for 20 minutes in the dark. TMB substrate (R&D Systems) was added after a further wash and left to develop in the dark for 20 minutes. Absorbance at 450 nm was read following the addition of 2 N sulphuric acid (‘stop’ solution) using a FLUOstar OPTIMA plate reader. Ang (1–7) concentration was interpolated from a standard curve generated by serially diluting recombinant human Ang (1–7) (5000–78.125 pg/ml). The assay showed minimal cross-reactivity with a number of closely related peptides, including Ang I, Ang II and Ang III.

### ACE-2 immunoperoxidase labelling

Formalin-fixed, paraffin-embedded tissue sections (7 μm) were cut and de-waxed prior to immunohistochemistry. Sections were pre-treated in trisodium citrate buffer (9 mM), pH 6, and microwaved for 5 minutes, left to stand for 5 minutes, and boiled for a further 5 minutes before being left to stand for 15 minutes at room temperature. Sections were then rinsed thoroughly and covered in horse serum blocking solution, rinsed again, and incubated overnight at room temperature with anti-ACE-2 antibody (0.05 μg/ml, ab15348; Abcam). Bound antibody was visualised using a biotinylated universal antibody followed by VECTASTAIN Elite ABC avidin-biotin complex kit (Vector Laboratories, Peterborough, UK) and a reaction with 0.01% H_2_O_2_. Specificity of the antibody was assessed by pre-adsorption of the ACE-2 antibody with a 250-fold molar excess of recombinant human ACE-2 protein (R&D Systems).

### Statistical analysis

Unpaired two-tailed *t* tests or analysis of variance (ANOVA) with Bonferroni’s post hoc analysis was used for comparisons between groups, and Pearson’s test was used to assess linear correlation with SPSS version 16 (SPSS, Chicago, IL, USA) and GraphPad Prism version 6 (GraphPad Software, La Jolla, CA, USA) software. *P* values <0.05 were considered statistically significant.

## Results

### ACE-2 enzyme activity is reduced in Alzheimer’s disease in association with increasing Aβ load and tau pathology

**Fig. 1:**
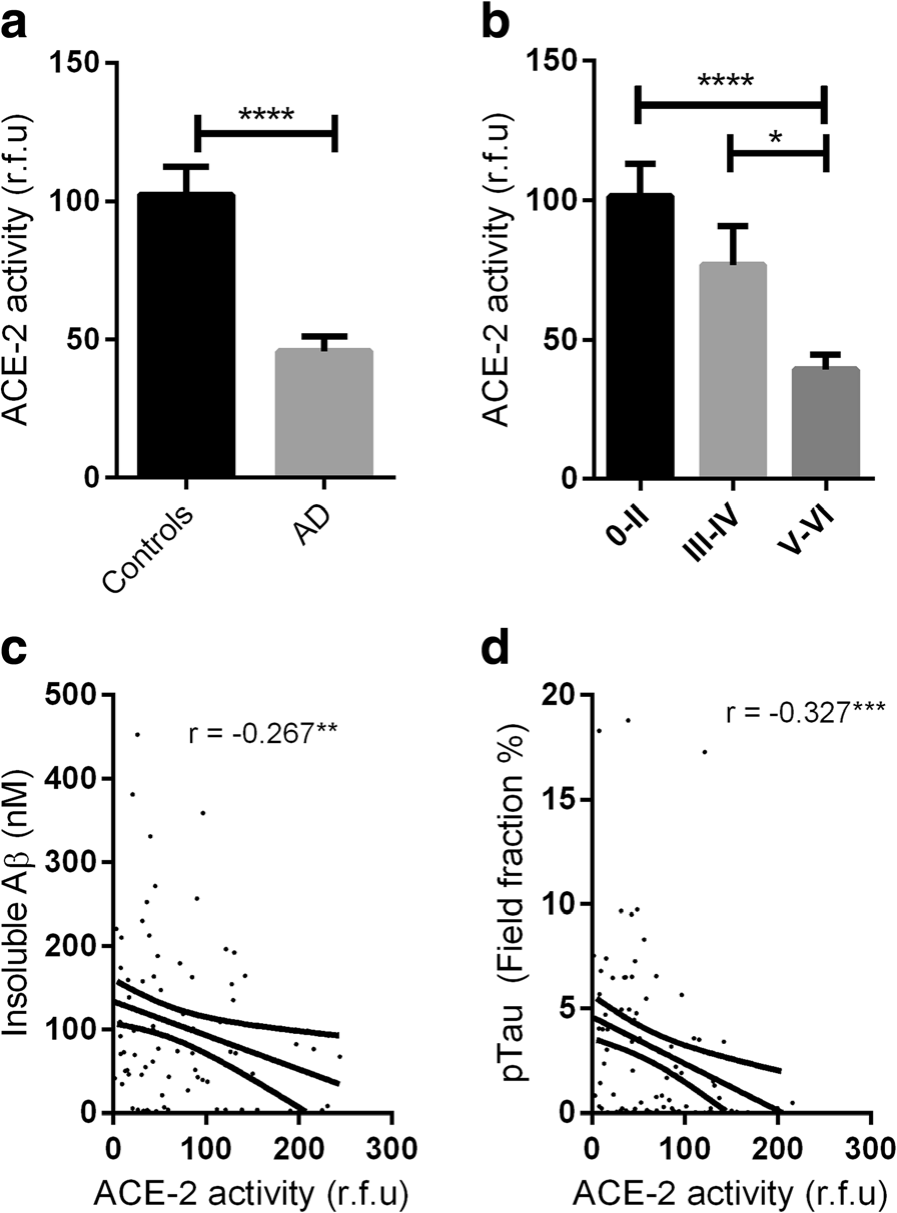
Angiotensin-converting enzyme 2 (ACE-2) activity is reduced in Alzheimer’s disease (AD). **a** Bar chart showing reduced ACE-2 activity in the mid-frontal cortex in AD (*n* = 90) compared with age-matched controls (*n* = 59) (*P* < 0.0001). **b** Bar chart showing reduced ACE-2 activity in relation to disease severity when all cases were combined and grouped according to Braak stage (0–II, II–IV, and V–VI) (*P* < 0.0001). Post hoc analysis revealed that ACE-2 activity was reduced in Braak tangle stages V–VI compared with stages 0–II and III-IV (﻿*P* < 0.0001 and *P < *0.05 respectively) and in Braak tangle stages III–IV compared with stages 0–II, but the difference was not statistically different. The bars indicate the mean value and SEM. **c** and **d** Scatterplots showing that ACE-2 activity was inversely correlated with insoluble amyloid-β (Aβ) load (measured by enzyme-linked immunosorbent assay) (*r* = −0.267, *P* < 0.01) and phosphorylated tau (p-tau) load (measured by field fraction analysis) (*r* = −0.327, *P* < 0.001). The *solid inner line* indicates the best-fit linear regression and the *outer lines* the 95% confidence intervals. *P < 0.05, **P < 0.01, ***P < 0.001, ****P < 0.0001. *rfu* Relative fluorescence units

ACE-2 activity was significantly reduced by approximately 50% in the mid-frontal cortex in AD compared with age-matched controls (*P* < 0.0001) (Fig. 1a). ACE-2 varied according to disease severity when the controls and AD cases were grouped and stratified into the following Braak tangle stage groups: 0–II, III–IV, and V-VI (*P* < 0.0001 by ANOVA). Post hoc analysis using the Bonferroni correction for multiple comparisons revealed that ACE-2 activity was significantly reduced in Braak tangle stages V–VI compared with stages 0–II (*P* < 0.0001) and stages III–IV (*P* < 0.05) (Fig. 1b). No difference was observed between Braak stages 0–II and stages III–IV.

In a combined AD and control cohort, ACE-2 activity correlated inversely with total insoluble Aβ levels (*r* = −0.267, *P* < 0.01) (Fig. 1c) but not with soluble Aβ (data not shown). ACE-2 correlated inversely with β-secretase activity (*r* = −0.277 *P* < 0.001) (Additional file 3: Figure S2). ACE-2 correlated inversely with p-tau load (*r* = 0.327, *P* < 0.01) (Fig. 1d).

### ACE-2 activity is reduced in relation to *APOE* and *ACE1* polymorphisms and CAA severity

**Fig. 2:**
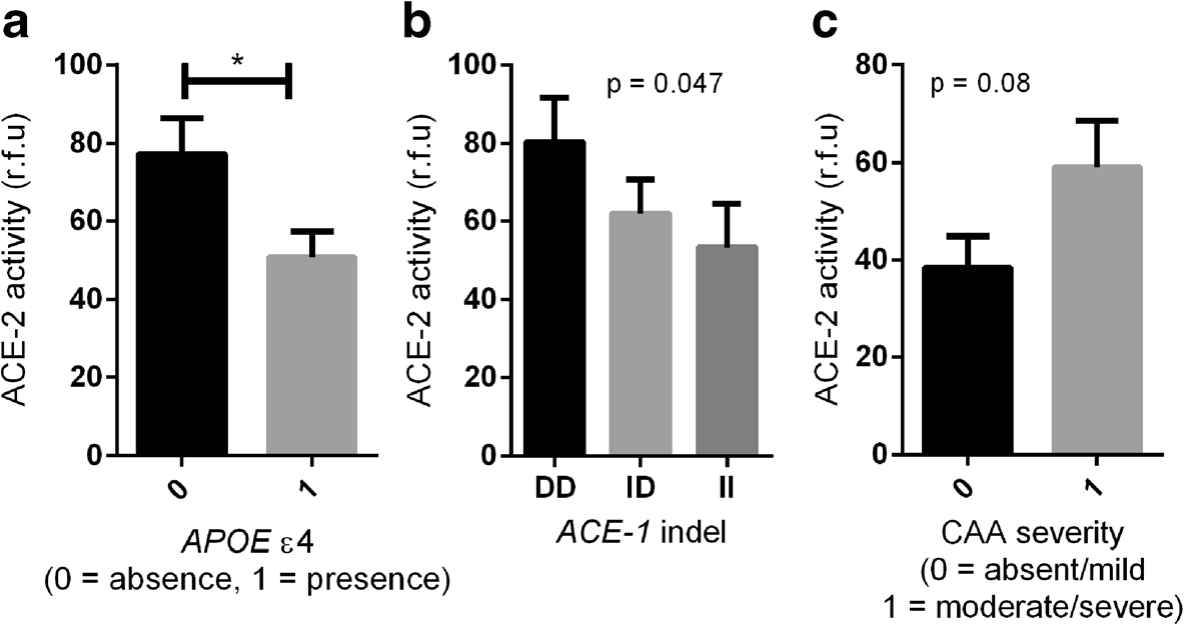
Angiotensin-converting enzyme 2 (ACE-2) activity is reduced in association with apolipoprotein E (*APOE*) ε4 and *ACE1* (rs1799752) indel polymorphism and increased in cerebral amyloid angiopathy (CAA). **a** Bar chart showing reduced ACE-2 activity in individuals with an *APOE* ε4 allele (*P* < 0.05). **b** Bar chart showing that ACE-2 activity varied according to *ACE1* ﻿indel polymorphism (*P* < 0.05), with a trend towards reduced ACE-2 activity in *ACE-1 * II homozygotes. **c** Bar chart showing elevated ACE-2 activity in moderate to severe CAA compared with absent to mild CAA, approaching significance (*P* = 0.08). The bars indicate the mean value and SEM. *P < 0.05. *rfu* Relative fluorescence units

ACE-2 activity was significantly lower in individuals possessing an *APOE* ε4 allele, an established genetic risk factor for sporadic AD [49], than in those without (*P* < 0.05) (Fig. 2a). ACE-2 activity also differed significantly between *ACE1* (rs1799752) indel genotypes (*P* < 0.05), with individuals who were homozygous II for *﻿ACE-1* (previously associated with increased risk for AD [17]) having the lowest ACE-2 activity, although post hoc analysis revealed that this did not reach statistical significance (Fig. 2b).

We assessed ACE-2 activity in relation to CAA severity and found, as for ACE-1 activity [4], a tendency, although not significant, towards increased ACE-2 activity in cases with moderate to severe CAA compared with absent to mild CAA (*P* = 0.08) (Fig. 2c).

### ACE-2 is inversely correlated with ACE-1, and the ratio of ACE-1 to ACE-2 is increased in Alzheimer’s disease

**Fig. 3:**
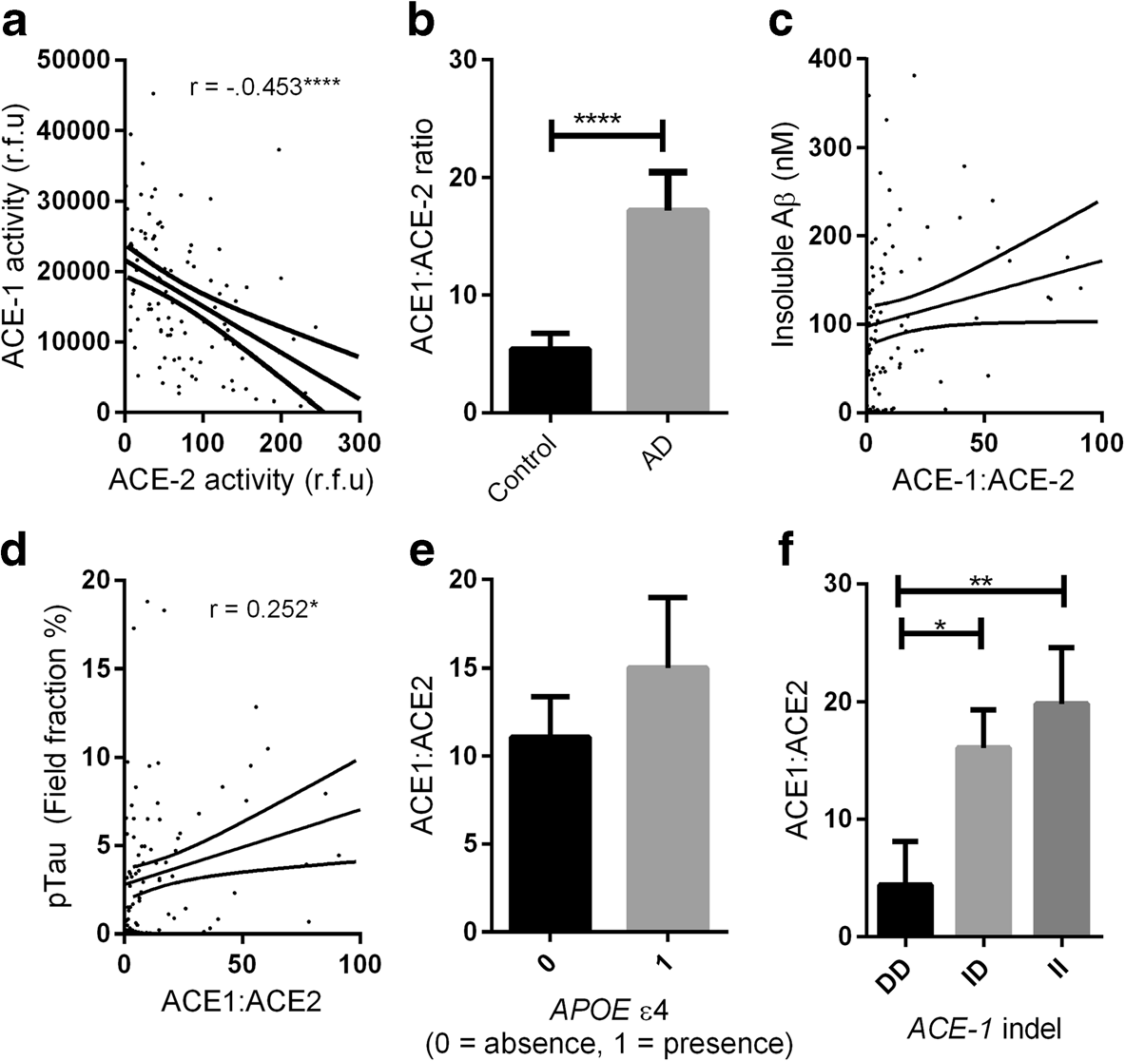
Angiotensin-converting enzyme 2 (ACE-2) activity is inversely correlated with ACE-1 activity, and the ACE-1/ACE-2 ratio is increased, in Alzheimer’s disease (AD). **a** Scatterplot showing a strong inverse relationship between ACE-1 and ACE-2 activity in mid-frontal cortex (*r* = −.453, *P* < 0.0001). The *inner solid line* indicates the best-fit linear regression and the *outer lines* the 95% confidence intervals. Each dot represents an individual brain. **b** Bar chart showing elevated ACE-1/ACE-2 ratio in AD (*P* < 0.0001). **c** and **d** Scatterplots showing positive correlation between the ACE-1/ACE-2 ratio and insoluble amyloid-β (Aβ) load (*r* = 0.199, *P* = 0.059) and p-tau load (*r* = 0.252, *P* < 0.05). **e** Bar chart showing a trend towards increased ACE-1/ACE-2 ratio in individuals who possessed an apolipoprotein E (*APOE*) ε4 allele. **f** Bar chart showing lower ACE:ACE-2 ratio in individuals who were homozygous DD for the *ACE1* (rs1799752) indel polymorphism compared with II (*P* < 0.01) and ID (*P* < 0.05). The bars indicate the mean value and SEM. *P < 0.05, **P < 0.01, ****P < 0.0001. *rfu* Relative fluorescence units

ACE-2 activity correlated inversely with ACE-1 activity in a combined AD and control cohort (*r* = −0.453, *P* > 0.0001) (Fig. 3a). The same pattern was observed and remained statistically significant when the control (*r* = −0.390, *P* < 0.05) and AD (*r* = −0.257, *P* < 0.05) groups were analysed separately.

Previous reports have suggested the ratio of ACE-1 to ACE-2 is a good proxy measure for the activation status of classical and regulatory RAS pathways [33]. With this in mind, we calculated the ACE-1/ACE-2 ratio for all cases and found that it was significantly increased in AD compared with controls (*P* > 0.0001) (Fig. 3b). The ACE-1/ACE-2 ratio also correlated positively with insoluble Aβ level, approaching significance (*r* = 0.199, *P* = 0.059) (Fig. 3c), and significantly with p-tau (*r* = 0.252, *P* < 0.05) (Fig. 3d). The ACE-1/ACE-2 ratio was increased in individuals possessing an *APOE* ε4 allele, approaching significance (*P* = 0.093) (Fig. 3e), and differed significantly according to *ACE1* (rs1799752) indel polymorphism (*P* < 0.01). Post hoc analysis revealed that the ratio was significantly higher in individuals with *ACE1* II (AD risk factor) than in DD (*P* < 0.01) and in ID than in DD (*P* < 0.05) (Fig. 3f).

### Ang II/Ang (1-7) ratio is increased in AD

**Fig. 4:**
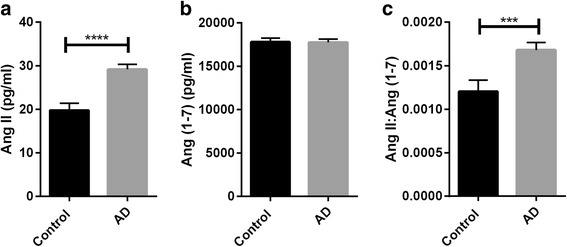
The ratio of angiotensin II (Ang II) to angiotensin (1–7) ﻿﻿(Ang ﻿(1-7)) (a proxy measure of ACE-2 activity) is increased, indicating reduced conversion of Ang II to Ang (1–7) in Alzheimer’s disease (AD). Bar charts showing **a** elevated Ang II levels in AD and **b** unchanged Ang (1–7) levels in AD compared with age-matched controls in mid-fontal cortex. **c** Bar chart showing the Ang II/Ang (1–7) ratio was significantly increased in AD (*P* < 0.001). The bars indicate the mean value and SEM. ***P < 0.001, ****P < 0.0001

Ang II levels were significantly increased in mid-frontal cortex in AD compared with age-matched controls (*P* < 0.0001) (Fig. 4a), whereas Ang (1–7) levels were unchanged (Fig. 4b). We calculated the Ang II/Ang (1–7) ratio (as a proxy indicator of ACE-2 activity) and found that the Ang II/Ang (1–7) ratio was significantly increased in AD (*P* > 0.001) (Fig. 4c). These data indicate that the conversion of Ang II to Ang (1–7) is likely to be reduced in AD because of lower ACE-2 activity.

### ACE-2 expression in human brain tissue

**Fig. 5:**
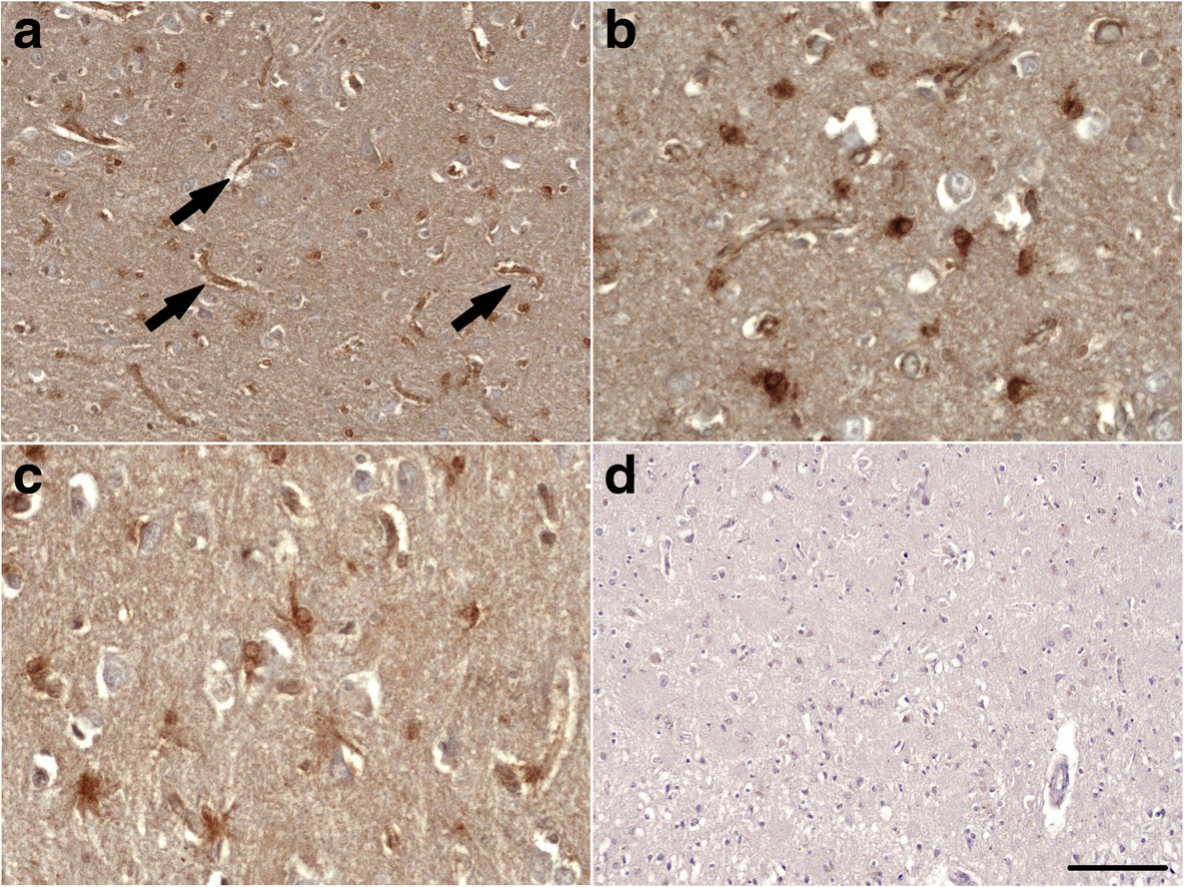
Angiotensin-converting enzyme 2 (ACE-2) expression in mid-frontal cortex in Alzheimer’s disease. **a** and **b** ACE-2 displayed strong capillary labelling (*black arrows*) and abundant perivascular labelling of larger arterioles (scale bar = 100 μm). Shown in **b** at higher magnification (scale bar = 50 μm). **b** and **c** ACE-2 was present in astrocytes (scale bar = 50 μm). **d** Pre-adsorption of ACE-2 antibody with recombinant human ACE-2 abolished labelling, confirming antibody specificity (scale bar = 100 μm)

ACE-2 was localised primarily to capillaries but also had a perivascular distribution around larger arterioles (Fig. 5a). ACE-2 labelled non-vascular cells that strongly resembled astrocytes (Fig. 5b and c). Labelling was not observed with pre-adsorption of the ACE-2 antibody with recombinant human ACE-2, demonstrating specificity of the antibody (Fig. 5d).

## Discussion

In the present study, we show that ACE-2 activity is reduced in post-mortem brain tissue in AD in association with increased Aβ and tau pathology. The reduction in ACE-2 was more pronounced in individuals carrying an *APOE* ε4 allele and in those who were homozygous II for the *ACE1* (rs1799752)﻿ indel polymorphism (both of which are suggested genetic risk factors for AD [17]). ACE-2 activity correlated inversely with ACE-1 activity (which we have previously shown to be increased in AD [4, 5]), and the ACE-1/ACE-2 ratio was higher in AD. Together, these data strongly suggest that reduced ACE-2 activity within the brain contributes to AD pathogenesis and is associated with increased activation of the central classical RAS axis.

The brain has its own intrinsic RAS [50–52], and we have shown in our previous studies that ACE-1, the rate-limiting enzyme in the production of Ang II, is overactive in AD [4, 5]. It is widely accepted that Ang II-mediated signalling via AT1R (commonly termed the *classical axis*) is overactive in AD and is associated with AD pathogenesis (reviewed in [1]). This view has been supported in various animal studies in which infusion of Ang II resulted in elevated plaque and tau pathology and significant cognitive impairment [2, 3]. Secondary observations in clinical trials and epidemiological studies have provided further evidence that RAS-targeting drugs that either block the production of Ang II or prevent AT1R-mediated signalling reduce the prevalence of AD [12–16], while cognitive performance is improved and pathology reduced, in animal models of AD [6–11]. Until recently, the prevailing view of the RAS in AD has been oversimplified because it has failed to consider the contribution of the other downstream RAS regulatory pathways within the brain.

In this study, we found reduced brain ACE-2 activity in AD, which supports a recent study showing lower peripheral serum ACE-2 levels in AD [37]. ACE-2 activity correlated inversely with parenchymal Aβ load and increased p-tau levels. We also observed a strong inverse relationship between ACE-2 and β-secretase activity, suggesting that ACE-2 may contribute in some way to regulating the amyloidogenic processing of APP. There are several possible mechanisms that link reduced ACE-2 activity to the pathogenesis of AD. Firstly, lower ACE-2 activity will, via a lower conversion of Ang II to Ang (1–7), result in elevated Ang II levels (as we have shown in this study). An increase in Ang II/Ang (1–7) ratio has commonly been reported in other chronic conditions associated with overactivation of the central axis [53]. Secondly, ACE-2 is primarily responsible for generating Ang (1–7) from Ang II [24, 54, 55], and subsequent Ang (1–7) activation of the Mas receptor counter-regulates the detrimental effects of the classical (ACE-1/Ang II/AT1R) axis [56–58] and has been linked with enhancing learning and memory processing [59, 60]. Lastly, ACE-2 has recently been shown to convert Aβ_43_, a highly amyloidogenic form of Aβ that seeds plaque formation [38], to Aβ_42_, which in turn is cleaved by ACE-1 to Aβ_40_ or, to a lesser extent, Aβ_41_, which have reduced toxicity [37]. Lower ACE-2 activity in AD may therefore promote the early deposition of Aβ_43_ and prevent downstream cleavage of Aβ_42_ by ACE-1.Together, these data suggest a putative protective role of the ACE-2/Ang (1–7)/Mas pathway, not only against the development of pathology but also against the decline in cognitive function, that is lost in AD.

Our findings indicate that the balance between the classical (ACE-1/Ang II/AT1R) axis and regulatory (ACE-2/Ang (1–7)/Mas) axis of RAS is disturbed in AD, as previously shown in various mouse models of cardiovascular disease [33] and diabetic nephropathy [53]. ACE-2 activity is reduced in AD and is inversely correlated with increasing ACE-1 activity, and the ACE-1/ACE-2 ratio is increased in AD in association with disease pathology. These findings support commonly observed traits in cardiac and renal pathologies showing that dysregulation of the ACE-2/Ang (1–7)/Mas pathway, including reduced ACE-2 activity, is associated with sustained hypertension mediated by overactivation of the classical axis (reviewed in [30, 61]). Despite the ratio of Ang II to Ang (1–7) (a proxy measure of ACE-2 activity) being increased in AD (i.e., reduced conversion of Ang II to Ang (1-7)), we did not observe an overall reduction in total Ang (1–7) in AD. This is inconsistent with a recent report showing reduced serum Ang (1–7) levels, rather than reduced ACE-2 activity, in senescence-accelerated mouse prone 8, a mouse model of sporadic AD (involving overexpression of APP). The authors observed that Ang (1–7) levels correlated inversely with Ang II and p-tau levels [39]. The reason for the discrepant findings between human and mouse brain tissue is unclear; however, both studies indicate that the ACE-2/Ang (1–7)/Mas pathway is dysregulated in AD and that further work is required to determine the exact contribution of each component of the pathway in AD.

Activation of the ACE-2/Ang (1–7)/Mas pathway, by inducing ACE-2 activity, or infusion of Ang (1–7) or a Mas receptor agonist, is protective in various experimental animal models of cardiovascular disease and is associated with a reduction of the classical RAS pathway (reviewed in [32, 61]). Neuronal overexpression of brain ACE-2 is also neuroprotective in a chronic hypertension mouse model (transgenic for renin and angiotensinogen that overproduces Ang II) following experimental induction of ischaemic stroke [34, 35, 62]. These protective effects were partially reversed in the presence of a Mas receptor antagonist, demonstrating the specificity of the ACE-2/Ang (1–7)/Mas pathway, and they have been shown to be mediated by counter-regulating the effects of Ang II-mediated reactive oxygen species production [63]. In AD, there is growing recognition that re-positioning of brain-penetrating ARBs and ACEIs may have clinical benefits in AD [64]. In addition to reducing the central pool of Ang II, ARBs and ACEIs might also exert their protective effects by preventing AT1R-mediated reduction in ACE-2 activity [65] that can be reversed by ARBs [27, 66–69]. ACE-2 activation is also associated with reduced ACE-1 activity [70] and with down-regulation of Ang II levels and AT1R expression [27, 65, 71–73]. These studies suggest that activation of ACE-2 may exert protective effects in AD above and beyond dampening RAS activation that the use of ACEIs and ARBs currently allow.

Lastly, we explored the distribution of ACE-2 within the mid-frontal and temporal cortices and found it to be localised predominantly within endothelial cells and smooth muscle cells of cerebral arteries, as previously reported [25]. Interestingly, as for ACE-1, we also observed extensive perivascular ACE-2 expression and found that ACE-2 activity was increased in individuals with moderate to severe CAA, as has previously been shown for ACE-1 [4]. We speculate that the sequential cleavage of Aβ_43_, first by ACE-2, and the subsequent cleavage of Aβ_42_ to Aβ_40_ (the predominant species in CAA [74]) by ACE-1, provides a potential mechanistic link with CAA. Further studies are required to determine the relationship between ACE-2 and CAA severity.

## Conclusions

These data indicate that reduced activity of the ACE-2/Ang (1–7)/Mas axis is strongly linked to overactivity of the classical RAS pathway and with AD-related pathology.

## Additional files

Additional file 1: Table S1. MRC identifiers for all cases. (DOC 80 kb)
Additional file 2: Figure S1. Scatterplot showing a strong positive correlation between two independent measures of ACE-2 activity in brain tissue samples. ACE-2 was measured using either a commercially available ACE-2 activity assay kit (SensoLyte® 390) or an ACE-2 fluorogenic peptide substrate (Mca-APK[Dnp]) in the presence of a selective ACE-2 inhibitor, MLN4760 (10 μM). The *solid inner line* indicates the best-fit linear regression, and the *outer lines* the 95% confidence intervals. Each point represents a separate brain. *****P* < 0.0001. (TIF 26 kb)
Additional file 3: Figure S2. Scatterplot showing an inverse relationship between ACE-2 activity and BACE-1 activity in a combined Alzheimer’s disease and age-matched control cohort. ACE-2 activity was measured using the SensoLyte® 390 ACE-2 activity assay kit, and BACE-1 activity was measured using the β-secretase specific fluorogenic substrate (Mca-SEVNLDAEFRK[Dnp]RR-NH2). The *inner solid line* indicates the best-fit linear regression, and the *outer lines* the 95% confidence intervals. Each point represents a separate brain. ****P* < 0.001. (TIF 25 kb)

## Abbreviations

ACE: Angiotensin-converting enzyme
ACEI: Angiotensin-converting enzyme inhibitors
AD: Alzheimer’s disease
Ang (1–7): Angiotensin (1–7) peptide
Ang (1–9): Angiotensin (1–9) peptide
Ang II: Angiotensin II peptide
ANOVA: Analysis of variance
APOE: Apolipoprotein E
APP: Amyloid precursor protein
ARB: Angiotensin II type 1 receptor blocker
AT1R: Angiotensin II type 1 receptor
Aβ: Amyloid-β
CAA: Cerebral amyloid angiopathy
D/D *ACE-1 *(rs1799752): Deletion/deletion polymorphism
ELISA: Enzyme-linked immunosorbent assay
FRET: Fluorescence resonance energy transfer
I/D *ACE-1* (rs1799752): Insertion/deletion polymorphism
I/I *ACE-1* (rs1799752): Insertion/insertion polymorphism
Mca: 7-Methoxycoumarin-4-yl-acetyl
MRC: Medical Research Council
MRC UK-BBN: Medical Research Council UK Brain Banks Network
p-tau: Phosphorylated tau
PM: Post-mortem
RAS: Renin-angiotensin system
rfu: Relative fluorescence units
TMB: 3,3′,5,5′-Tetramethylbenzidine
