# Supplementary material for: Angiotensin-converting enzyme 2 is reduced in Alzheimer’s disease in association with increasing amyloid-β and tau pathology
Source: Alzheimers Res Ther. 2016 Nov 25;8:50. doi: 10.1186/s13195-016-0217-7 (PMC5123239; doi:10.1186/s13195-016-0217-7)
Supplement: Additional file 1: Table S1. — MRC identifiers for all cases. (DOC 80 kb) [file 13195_2016_217_MOESM1_ESM.doc]

Table S1. MRC identifiers for all cases

| **Diagnosis** | **MRC ID** | **MRC ID** |
| --- | --- | --- |
| Control | BBN_8639 | BBN_8898 |
| Control | BBN_8644 | BBN_8923 |
| Control | BBN_8651 | BBN_8949 |
| Control | BBN_8671 | BBN_8956 |
| Control | BBN_8682 | BBN_8957 |
| Control | BBN_8684 | BBN_8961 |
| Control | BBN_8691 | BBN_8964 |
| Control | BBN_8700 | BBN_8966 |
| Control | BBN_8702 | BBN_8980 |
| Control | BBN_8703 | BBN_8983 |
| Control | BBN_8706 | BBN_9012 |
| Control | BBN_8707 | BBN_9016 |
| Control | BBN_8708 | BBN_9028 |
| Control | BBN_8709 | BBN_9038 |
| Control | BBN_8712 | BBN_9086 |
| Control | BBN_8714 | BBN_9092 |
| Control | BBN_8717 | BBN_9217 |
| Control | BBN_8722 | BBN_9256 |
| Control | BBN_8723 | BBN_9292 |
| Control | BBN_8725 | BBN_9299 |
| Control | BBN_8728 | BBN_9311 |
| Control | BBN_8731 |  |
| Control | BBN_8732 |  |
| Control | BBN_8735 |  |
| Control | BBN_8739 |  |
| Control | BBN_8741 |  |
| Control | BBN_8749 |  |
| Control | BBN_8751 |  |
| Control | BBN_8756 |  |
| Control | BBN_8757 |  |
| Control | BBN_8759 |  |
| Control | BBN_8768 |  |
| Control | BBN_8770 |  |
| Control | BBN_8776 |  |
| Control | BBN_8779 |  |
| Control | BBN_8835 |  |
| Control | BBN_8883 |  |
| Control | BBN_8888 |  |

| **Diagnosis** | **MRC ID** | **MRC ID** | **MRC ID** |
| --- | --- | --- | --- |
| AD | BBN_8819 | BBN_9114 | BBN_9256 |
| AD | BBN_8825 | BBN_9119 | BBN_9298 |
| AD | BBN_8834 | BBN_9122 | BBN_9301 |
| AD | BBN_8839 | BBN_9123 | BBN_9303 |
| AD | BBN_8841 | BBN_9125 | BBN_9304 |
| AD | BBN_8842 | BBN_9132 | BBN_9309 |
| AD | BBN_8848 | BBN_9136 | BBN_9310 |
| AD | BBN_8852 | BBN_9156 | BBN_9315 |
| AD | BBN_8853 | BBN_9162 | BBN_9320 |
| AD | BBN_8857 | BBN_9165 | BBN_9323 |
| AD | BBN_8870 | BBN_9167 |  |
| AD | BBN_8870 | BBN_9179 |  |
| AD | BBN_8871 | BBN_9182 |  |
| AD | BBN_8885 | BBN_9123 |  |
| AD | BBN_8886 | BBN_9188 |  |
| AD | BBN_8892 | BBN_9189 |  |
| AD | BBN_8905 | BBN_9193 |  |
| AD | BBN_8906 | BBN_9194 |  |
| AD | BBN_8912 | BBN_9198 |  |
| AD | BBN_8915 | BBN_9200 |  |
| AD | BBN_8917 | BBN_9201 |  |
| AD | BBN_8918 | BBN_9205 |  |
| AD | BBN_8930 | BBN_9207 |  |
| AD | BBN_8947 | BBN_9209 |  |
| AD | BBN_8954 | BBN_9212 |  |
| AD | BBN_8958 | BBN_9221 |  |
| AD | BBN_8969 | BBN_9222 |  |
| AD | BBN_8997 | BBN_9243 |  |
| AD | BBN_9005 | BBN_9248 |  |
| AD | BBN_9026 | BBN_9259 |  |
| AD | BBN_9031 | BBN_9261 |  |
| AD | BBN_9050 | BBN_9263 |  |
| AD | BBN_9052 | BBN_9265 |  |
| AD | BBN_9061 | BBN_9269 |  |
| AD | BBN_9076 | BBN_9274 |  |
| AD | BBN_9095 | BBN_9275 |  |
| AD | BBN_9102 | BBN_9284 |  |
| AD | BBN_9106 | BBN_9291 |  |
| AD | BBN_9112 | BBN_9293 |  |
| AD | BBN_9113 | BBN_9295 |  |
